# Supplementary material for: High entropy alloy property predictions using a transformer-based language model
Source: Sci Rep. 2025 Apr 7;15:11861. doi: 10.1038/s41598-025-95170-z (PMC11977270; doi:10.1038/s41598-025-95170-z)
Supplement: Supplementary file 1 — Supplementary Material 1 [file 41598_2025_95170_MOESM1_ESM.docx]

**Appendix A**

**General Symbols**

- $S$ : A chemical composition string (e.g., "Co1.2 Fe0.8 Ni1").
- $\mathcal{A}$ : The set of all chemical element symbols.
- $N$ : Number of samples in the dataset or number of Transformer layers, depending on context.
- $L$ : Length of the input token sequence.
- $h$ : Number of attention heads in the Transformer model.
- d: Dimensionality of the embedding space.
- $d_{k}$ : Dimensionality of the query and key vectors, typically $d_{k}=d/h$.
- $d_{\mathrm{ff}}$ : Dimensionality of the feed-forward network's inner layer.
- $\sigma$ : Activation function (e.g., GELU, ReLU) or standard deviation, depending on context.
- $\mu$ : Mean value, often of a feature or target variable.
- $\eta$ : Learning rate used in optimization.
- $\lambda$ : Weight decay coefficient for regularization.
- $\theta$ : Model parameters.
- $\gamma$ : Gradient clipping threshold.

**Data Preprocessing Symbols**

- $E_{i}$ : The $i$-th chemical element symbol extracted from a composition.
- $f_{i}$ : Fractional abundance (stoichiometric coefficient) of element $E_{i}$.
- $\mathcal{E}(S)$ : Set of element-fraction pairs extracted from composition $S$ :

$$\mathcal{E}(S)=\left\{ \left( E_{i},f_{i} \right)\mid i=1,\ldots,N_{S} \right\}$$

- $N_{S}$ : Number of unique elements in composition $S$.
- $\mathcal{E}^{'}(S)$ : Alphabetically sorted set of element-fraction pairs from $S$.
- $T_{i}$ : Token formed by concatenating $E_{i}$ and $f_{i}$ :

$$T_{i}=E_{i}\circ f_{i}$$

- $\mathcal{T}(S)$ : Sequence of tokens for composition $S$ :

$$\mathcal{T}(S)=\left[ T_{1},T_{2},\ldots,T_{N_{S}} \right]$$

- $x_{j}$ : The $j$-th numerical feature (e.g., temperature, pressure).
- $x_{j}:$ Normalized numerical feature:

$$x_{j}=\frac{x_{j}-\mu_{x_{j}}}{\sigma_{x_{j}}}$$

- $C_{S}$ : Combined features for sample $S$, including tokenized elements and numerical features:

$$C_{S}=\mathcal{T}(S)\cup\left\{ x_{1},x_{2},\ldots,x_{M} \right\}$$

- $y$ : Original target variable (e.g., hardness, ultimate tensile strength).
- $y$ : Normalized target variable:

$$y=\frac{y-\mu_{\text{train }}}{\sigma_{\text{train }}}$$

- $\mu_{\text{train }},\sigma_{\text{train }}:$ Mean and standard deviation of the target variable in the training set.

**Tokenization and Embedding Symbols**

- $\mathbf{t}=\left[ t_{1},t_{2},\ldots,t_{L} \right]$ : Tokenized input sequence after applying BERT tokenizer.
- V: Vocabulary of the tokenizer.
- $\mathrm{ID}\left( t_{i} \right)$ : Vocabulary index of token $t_{i}$.
- $\mathbf{t}_{\text{ids }}=\left[ \mathrm{ID}\left( t_{1} \right),ID\left( t_{2} \right),\ldots,ID\left( t_{L} \right) \right]$ : Sequence of token IDs.
- E: Token embedding matrix of size $|V|\times d$.
- $\mathbf{1}_{t_{i}}$ : One-hot encoding vector for token $t_{i}$.
- $\mathbf{e}_{t_{i}}$ : Token embedding for $t_{i}$ :

$$\mathbf{e}_{t_{i}}=\mathbf{E}\cdot\mathbf{1}_{t_{i}}$$

- $\mathbf{E}_{t}=\left[ \mathbf{e}_{t_{1}},\mathbf{e}_{t_{2}},\ldots,\mathbf{e}_{t_{L}} \right]$ : Sequence of token embeddings.
- $\mathbf{e}_{\mathrm{pos}_{i}}$ : Positional embedding for position $i$.
- $\mathbf{E}_{\mathrm{pos}}=\left[ \mathbf{e}_{\mathrm{pos}_{1}},\mathbf{e}_{\mathrm{pos}_{2}},\ldots,\mathbf{e}_{\mathrm{pos}_{L}} \right]$ : Sequence of positional embeddings.
- $\mathbf{e}_{\text{seg }}$ : Segment embedding (constant vector for all positions in single-sequence tasks).
- $\mathbf{E}_{\text{seg }}=\left[ \mathbf{e}_{\text{seg }},\mathbf{e}_{\text{seg }},\ldots,\mathbf{e}_{\text{seg }} \right]:$ Sequence of segment embeddings.
- $\mathbf{H}^{(0)}$ : Input embeddings to the Transformer encoder:

$$\mathbf{H}^{(0)}=\mathbf{E}_{t}+\mathbf{E}_{\mathrm{pos}}+\mathbf{E}_{\mathrm{seg}}$$

- $\mathbf{H}^{(l)}$ : Hidden states at layer $l$ of the Transformer encoder.

**Transformer Encoder Symbols**
**Multi-Head Self-Attention**

- $\mathbf{Q}_{i},\mathbf{K}_{i},\mathbf{V}_{i}$ : Query, key, and value vectors at position $i$ :

$$\begin{matrix} \mathbf{Q}_{i} & =\mathbf{W}_{Q}\mathbf{H}_{i}^{(l-1)} \\ \mathbf{K}_{i} & =\mathbf{W}_{K}\mathbf{H}_{i}^{(l-1)} \\ \mathbf{V}_{i} & =\mathbf{W}_{V}\mathbf{H}_{i}^{(l-1)} \end{matrix}$$

- $\mathbf{W}_{Q},\mathbf{W}_{K},\mathbf{W}_{V}\in\mathbb{R}^{d\times d_{k}}$ : Projection matrices for queries, keys, and values.
- $\mathbf{Q}_{i}^{(h)},\mathbf{K}_{i}^{(h)},\mathbf{V}_{i}^{(h)}$ : Query, key, and value vectors for head $h$.
- $\alpha_{ij}^{(h)}:$ Attention weight from position $i$ to position $j$ in head $h$ :

$$\alpha_{ij}^{(h)}=softmax\left( \frac{\mathbf{Q}_{i}^{(h)}\cdot\left( \mathbf{K}_{j}^{(h)} \right)^{\top}}{\sqrt{d_{k}}} \right)$$

- $\mathbf{Z}_{i}^{(h)}$ : Output of attention head $h$ at position $i$ :

$$\mathbf{Z}_{i}^{(h)}=\sum_{j=1}^{L} \alpha_{ij}^{(h)}\mathbf{V}_{j}^{(h)}$$

- $\mathbf{Z}_{i}$ : Concatenated output of all attention heads at position $i$ :

$$\mathbf{Z}_{i}=\mathbf{W}_{O}\left[ \mathbf{Z}_{i}^{(1)}\left\| \mathbf{Z}_{i}^{(2)} \right\|\ldots\|\mathbf{Z}_{i}^{(h)} \right]$$

- $\mathbf{W}_{O}\in\mathbb{R}^{d\times d}$ : Output projection matrix after concatenation.
- II: Concatenation operator for vectors.
- $\mathbf{H}_{i}^{(l)}$ : Output after residual connection and layer normalization:

$$\mathbf{H}_{i}^{(l)}=LayerNorm\left( \mathbf{H}_{i}^{(l-1)}+\mathbf{Z}_{i} \right)$$

**Position-Wise Feed-Forward Network**

- $\mathbf{F}_{i}^{(l)}$ : Output of the feed-forward network at layer $l$, position $i$ :

$$\mathbf{F}_{i}^{(l)}=\sigma\left( \mathbf{W}_{1}\mathbf{H}_{i}^{(l)}+\mathbf{b}_{1} \right)\mathbf{W}_{2}+\mathbf{b}_{2}$$

- $\mathbf{W}_{1}\in\mathbb{R}^{d\times d_{d}},\mathbf{W}_{2}\in\mathbb{R}^{d_{II}\times d}$ : Weights of the feed-forward network.
- $\mathbf{b}_{1}\in\mathbb{R}^{d_{\pi}},\mathbf{b}_{2}\in\mathbb{R}^{d}$ : Biases of the feed-forward network.
- $\sigma$ : Activation function (e.g., GELU, ReLU).
- $\mathbf{H}_{i}^{(l)}$ : Output after the second residual connection and layer normalization:

$$\mathbf{H}_{i}^{(l)}=LayerNorm\left( \mathbf{H}_{i}^{(l)}+\mathbf{F}_{i}^{(l)} \right)$$

**Regression Head Symbols**

- $\mathbf{h}_{\text{CLS }}=\mathbf{H}_{1}^{(N)}$ : Final hidden state of the [CLS] token after $N$ Transformer layers.
- $\mathbf{W}_{\text{reg }}\in\mathbb{R}^{d}$ : Weights of the regression head.
- $b_{\text{reg }}\in\mathbb{R}$ : Bias term of the regression head.
- $y$ : Predicted normalized target value:

$$y=\mathbf{W}_{\mathrm{reg}}^{\top}\mathbf{h}_{\mathrm{CLS}}+b_{\mathrm{reg}}$$

- $y_{i}^{\text{(orig) }}$ : Predicted target value in original scale for sample $i$ :

$$y_{i}^{(\text{orig })}=y_{i}\sigma_{\text{train }}+\mu_{\text{train }}$$

**Loss Function and Evaluation Metrics**

- $\mathcal{L}(\theta)$ : Mean Squared Error (MSE) loss function:

$$\mathcal{L}(\theta)=\frac{1}{N}\sum_{i=1}^{N} \left( y_{i}-y_{i} \right)^{2}$$

- MSE: Mean Squared Error metric:

$$MSE=\frac{1}{N_{\mathrm{val}}}\sum_{i=1}^{N_{\mathrm{ral}}} \left( y_{i}-y_{i} \right)^{2}$$

- MAE: Mean Absolute Error metric:

$$MAE=\frac{1}{N_{\mathrm{val}}}\sum_{i=1}^{N_{\mathrm{val}}} \left| y_{i}-y_{i} \right|$$

- $R^{2}$ : Coefficient of determination:

$$R^{2}=1-\frac{\sum_{i=1}^{N_{\mathrm{vel}}} \left( y_{i}-y_{i} \right)^{2}}{\sum_{i=1}^{N_{\mathrm{vel}}} \left( y_{i}-y_{\mathrm{val}} \right)^{2}}$$

- $y_{\mathrm{val}}$ : Mean of the true normalized target values in the validation set.

**Residuals and Analysis Symbols**

- $r_{i}$ : Residual for sample $i$ :

$$r_{i}=y_{i}-y_{i}^{\text{(orig) }}$$

- M: Attention mask used to indicate valid tokens versus padding tokens.
- LayerNorm( $\mathbf{x}$ ): Layer normalization function applied to vector $\mathbf{x}$ :

$$LayerNorm(\mathbf{x})=\frac{\mathbf{x}-\mu_{x}}{\sigma_{x}}\cdot\gamma+\beta$$

- $\mu_{x},\sigma_{x}$ : Mean and standard deviation of components of $\mathbf{x}$.
- $\gamma,\beta$ : Learnable scaling and shifting parameters.

**Optimization Symbols**

- $\nabla_{\theta}\mathcal{L}(\theta)$ : Gradient of the loss function with respect to model parameters.
- $\theta_{t}$ : Model parameters at iteration $t$.
- $\theta_{t+1}$ : Updated model parameters after applying optimization step.
- $\lambda\theta$ : Weight decay term for regularization.
- $\frac{\partial\theta_{1}}{\partial\theta}$ : Gradient of the prediction with respect to model parameters.

**Miscellaneous Symbols**

- $\mathrm{clip}\left( \theta_{t},-\gamma,\gamma\right)$ : Gradient clipping operation to keep parameters within $[-\gamma,\gamma]$.
- softmax( $\cdot$ : Softmax function applied to attention scores to obtain attention weights.
- FFN $(\cdot)$ : Position-wise feed-forward network function.
- $\mathbf{H}_{i}^{(l-1)}$ : Hidden state at layer $l-1$, position $i$.
- $\mathbf{H}^{(N)}$ : Final hidden states after $N$ Transformer layers.

Note: All vectors are assumed to be column vectors unless specified otherwise. Matrices and vectors are represented in boldface, while scalars are in regular typeface. The operators and functions are standard in linear algebra and machine learning contexts.
